# Impact of prophylactic donor heart tricuspid valve annuloplasty on outcomes in heart transplantation

**DOI:** 10.1186/s13019-023-02396-x

**Published:** 2023-10-12

**Authors:** Hidefumi Nishida, Valluvan Jeevanandam, Christopher Salerno, Atsushi Nemoto, Tae Song, David Onsager, Ann Nguyen, Jonathan Grinstein, Bow Chung, Nitasha Sarswat, Gene Kim, Sean Pinney, Takeyoshi Ota

**Affiliations:** 1 Department of Surgery, Section of Cardiac Surgery, The University of Chicago Medicine, 5841S Maryland Avenue, MC5040, Chicago, IL 60637 USA; 2 Department of Medicine, Section of Cardiology, The University of Chicago Medicine, Chicago, IL USA

**Keywords:** Heart transplantation, Tricuspid valve annuloplasty, Transplantation

## Abstract

**Background:**

Tricuspid regurgitation(TR) following heart transplantation could adversely affect clinical outcomes. In an effort to reduce the incidence of TR, prophylactic donor heart tricuspid valve annuloplasty has been performed during heart transplantation in our institution. We assessed early and long-term outcomes.

**Methods:**

Between August 2011 and August 2021, 349 patients who underwent prophylactic tricuspid valve annuloplasty were included. Tricuspid valve annuloplasty was performed using the DeVega annuloplasty technique. The clinical outcomes of the interests included complete atrioventricular block requiring pacemaker implantation, the occurrence of significant TR(defined as moderate or greater), and survival. Long-term survival was compared in patients with and without significant TR using the Kaplan-Meier method. The Cox proportional hazards regression with time-dependent covariate analysis was used to see if significant TR affected the long-term survival.

**Results:**

There was one patient(0.3%) who required pacemaker implantation for complete atrioventricular block. No patients developed tricuspid valve stenosis that required intervention. Significant TR developed in 31 patients(8.9%) during the follow-up period. The survival rate of patients who developed significant TR was significantly lower than that of those who did not(log rank < 0.01). Significant TR was associated with the long-term mortality(HR2.92, 95%CI 1.47–5.82, p < 0.01).

**Conclusions:**

Prophylactic donor heart tricuspid valve annuloplasty has the potential to reduce the occurrence of significant TR and can be performed safely. The significant TR that developed in patients with prophylactic annuloplasty negatively affected survival and was an independent predictor of long-term mortality.

## Introduction

Recent advances in medicine and technology have contributed to improving the clinical outcomes of orthotopic heart transplantation (OHT). However, the development of tricuspid regurgitation (TR) after OHT remains an important complication and can affect clinical outcomes. Incidence of TR after OHT has been reported in the range from 20–40% [1–4]. The causes of TR after OHT are multifactorial including persistent pulmonary hypertension, allograft dysfunction, the tricuspid valve geometry change from the surgical techniques (i.e. the biatrial technique), and structural injuries during catheter-based biopsies [1–3, 6, 7]. It has been reported that TR after OHT is associated with adverse clinical outcomes including right side heart failure, renal dysfunction, and survival [1, 2, 8–10]. In an effort to reduce the incidence of TR after OHT, we perform a prophylactic tricuspid annuloplasty during OHT in our institution. The purpose of this study was to investigate the impact of prophylactic tricuspid valve annuloplasty (TVA) in OHT on early and late clinical outcomes.

## Patients and methods

### Patients and study design

Between August 2011 and August 2021, 350 patients underwent OHT at the University of Chicago Medicine. One patient who did not receive TVA was excluded from this study. As a result, the cohort included 349 patients.

This is a retrospective observational study. The definition of significant TR is moderate or greater TR evaluated by transthoracic echocardiography during the follow-up period. Because TR can fluctuate, we also investigated patients with persistent significant TR (defined as significant TR that was consistent for more than two months). The cohort was divided into two groups: patients who developed significant TR after OHT during follow-up (TR group) and those who did not (non-TR group). Perioperative data and late clinical outcomes were reviewed. We evaluated the impact of significant TR on long-term survival using multivariate analyses. All data were retrospectively reviewed from each patient’s electric medical records. The mean follow-up period was 43.8 ± 32.9 months after OHT.

### DeVega annuloplasty

The DeVega annuloplasty technique [11] was used for TVA in donor hearts in all cases. They were performed on the back table before implantation. Briefly, the tricuspid valve was exposed through the inferior cava. A double layer of a pledgeted 2 − 0 polypropylene suture was started at the fibrous trigone in the vicinity of the antero-septal commissure, and continued down to the posterior extremity of the septal portion of the annulus in a clockwise direction, through the anterior and posterior portions of the annulus. The suture needle penetrated at a depth of 1 to 2 mm, in bites approximately 5 to 6 mm long. The second suture also followed the same path as the second suture line intercalated that of the first one. The sutures were tied over a pledget. The degree of annulus narrowing was controlled between 26 and 30 mm, depending on the donor heart size.

### Echocardiographic evaluation of TR

A degree of TR was assessed by transthoracic echocardiography during follow-up. Significant TR was defined as valvular regurgitation of moderate or greater. Valvular regurgitation was graded according to the guidelines of the American Society of Echocardiography [12]. Postoperative echocardiography was performed for all patients, and routine follow-up echocardiography was conducted at 1 month, 3 months, 6 months, 12 months, and annually. Out of total patient population, 348 patients (99.7%) underwent at least one follow-up echocardiography.

### Statistical analyses

All data analyses were performed with JMP 11.0 software (SAS Institute Inc, Cary, NC, USA). Data were expressed as means ± standard deviations or median and ranges for continuous variables and as numbers (percentages) for categorical variables. Comparisons of continuous variables were tested with the unpaired t test or Wilcoxon test, and comparisons of categorical variables were tested with the chi-square test or Fisher’s exact test. Long-term survival and freedom from significant TR were analyzed using the Kaplan-Meier analysis and log-rank test. As for right ventricle function evaluations, we calculated pulmonary artery pulsatility index (PAPi) = (systolic pulmonary artery pressure – diastolic pulmonary artery pressure) divided by central venous pressure (CVP) as well as CVP/pulmonary artery wedge pressure (PAWP) ratio.

The association between the development of significant TR and long-term survival was analyzed with the Cox proportional hazard regression with time-dependent covariate analysis. We included covariates which were rationally considered to be related with long-term survival such as age, high-grade rejection (grade2 or higher), significant TR, and history of diabetes mellitus.

## Results

### Preoperative data

Preoperative characteristics are detailed in Table 1. Overall, the mean age was 52.9 ± 13.3 years and 266 patients (76.2%) were male. One hundred ninety-one patients (55.0%) had hypertension and 102 patients (29.4%) had diabetes mellitus. Two hundred forty-four patients (69.9%) had the non-ischemic cardiomyopathy. The mean left ventricle ejection fraction (EF) was 24.2 ± 10.9%. There were no significant differences between TR group and Non-TR group in hemodynamic parameters.

**Table 1:** Preoperative characteristics

	All (n = 349)	Non-TR group (n = 318)	TR group (n = 31)	p
Mean age (years)	52.9 ± 13.3	53.0 ± 13.4	51.0 ± 12.2	0.41
Male	266 (76.2%)	241 (75.8%)	25 (80.6%)	0.54
Height (cm)	174.5 ± 10.3	174.4 ± 10.4	176.3 ± 9.8	0.32
Weight (kg)	86.9 ± 18.9	86.0 ± 18.9	96.9 ± 16.8	< 0.01
Body mass index	28.8 ± 5.1	28.1 ± 5.0	31.3 ± 4.9	< 0.01
Hypertension	191 (55.0%)	167 (52.5%)	24 (77.4%)	< 0.01
Dyslipidemia	112 (32.3%)	103 (32.4%)	9 (2.9%)	0.68
Diabetes mellitus	102 (29.4%)	92 (28.9%)	10 (32.3%)	0.72
LVAD	81 (23.2%)	72 (22.6%)	9 (29.0%)	0.43
Nonischemic cardiomyopathy	244 (69.9%)	225 (70.8%)	19 (61.3%)	0.30
Previous cardiac surgery	168 (48.1%)	149 (46.9%)	19 (61.3%)	0.12
Creatinine (mg/dl)	1.48 ± 1.1	1.44 ± 1.0	1.48 ± 0.7	0.81
LVDd (mm)	65.9 ± 12.8	65.7 ± 12.9	70.1 ± 12.1	0.15
Ejection fraction (%)	24.2 ± 10.9	24.2 ± 11.2	24.4 ± 6.7	0.92
CVP (mmHg)	10.9 ± 6.6	10.9 ± 6.6	9.7 ± 5.7	0.32
Mean PAP (mmHg)	30.4 ± 11.2	30.3 ± 11.2	30.3 ± 10.7	0.98
PAWP (mmHg)	20.3 ± 9.3	20.2 ± 9.3	19.5 ± 8.7	0.68
PAPi	2.84 ± 2.9	2.76 ± 2.73	3.83 ± 4.4	0.07
CVP/PAWP	0.56 ± 0.3	0.56 ± 0.3	0.51 ± 0.4	0.37
CI (L/min/m^2^)	2.35 ± 0.7	2.37 ± 0.7	2.15 ± 0.5	0.09

LVAD: left ventricle assist device, LVDd: left ventricular end-diastolic diameter, CVP: central venous pressure, PAP: pulmonary artery pressure, PAWP: pulmonary artery wedge pressure, CI: cardiac index

### Intraoperative data

Intraoperative data are shown in Table 2. Overall, the total heart transplantation technique for pulmonary vein anastomoses was used in 106 patients (30.4%). The donor heart ischemic time was 238.2 ± 60.5 min and cross clamp time was 150.8 ± 38.7 min. There were no significant differences between two groups in the anastomosis techniques, cardiopulmonary bypass time, cross clamp time, implant time (defined as a duration from the first anastomosis stich on the donor heart to declamping the aorta), reperfusion time and donor heart ischemic time.

**Table 2:** Operative data and early outcomes after heart transplantation

Variables	All (n = 349)	Non-TR group (n = 318)	TR group (n = 31)	p
Operative procedures				
Bicaval anastomosis	243 (69.6%)	221 (69.5%)	22 (71.0%)	0.97
Total anastomosis	106 (30.4%)	97 (30.5%)	9 (29.0%)	
Ischemic time (min)	238.2 ± 60.5	237.4 ± 60.7	246.0 ± 60.3	0.47
Cardiopulmonary bypass time (min)	202.1 ± 60.7	202.4 ± 61.5	197.4 ± 54.3	0.67
Cross clamp time (min)	150.8 ± 38.7	150.8 ± 39.1	150.1 ± 36.9	0.92
Implant time (min)	89.7 ± 19.4	89.9 ± 19.2	87.3 ± 22.2	0.49
Reperfusion time (min)	33.3 ± 18.5	33.4 ± 18.3	32.4 ± 21.1	0.78
Early outcomes				
In hospital mortality	11 (3.2%)	11 (3.5%)	0 (0%)	0.15
ECMO	27 (7.7%)	23 (7.2%)	4 (12.9%)	0.30
Hemodialysis	27 (7.7%)	20 (6.3%)	7 (22.6%)	< 0.01
Re-exploration	33 (9.5%)	29 (9.1%)	4 (12.9%)	0.51
Stroke	14 (4.0%)	13 (4.1%)	1 (3.2%)	0.81
CVP (mmHg)	8.3 ± 4.5	8.0 ± 4.4	10.8 ± 5.5	< 0.01
Mean PAP (mmHg)	24.6 ± 7.2	24.3 ± 7.2	27.6 ± 6.6	0.02
PCWP (mmHg)	15.7 ± 5.7	15.4 ± 5.6	18.5 ± 5.6	< 0.01
PAPi	3.1 ± 2.5	3.17 ± 2.6	2.25 ± 1.1	0.06
CVP/PAWP	0.52 ± 0.2	0.51 ± 0.2	0.60 ± 0.2	< 0.01
CI (L/min/m^2^)	3.1 ± 0.7	3.1 ± 0.7	3.0 ± 0.6	0.40

ECMO: extracorporeal membranous oxygenation, CVP: central venous pressure, PAP: pulmonary artery pressure, PAPi: pulmonary artery pulsatile index, PAWP: pulmonary artery wedge pressure, CI: cardiac index

### Early clinical outcomes after heart transplantation

Early clinical outcomes are summarized in Table 2. Overall, hospital mortality was 3.2% (11/349). The causes of deaths were multiple organ failure in seven patients, respiratory failure in two patients, acute pancreatitis in one patient, and sepsis in one patient. Postoperative complications included re-exploration for bleeding in 33 patients (9.5%), extracorporeal membranous oxygenation (ECMO) requirement in 27 patients (7.7%), acute renal failure requiring hemodialysis in 27 patients (7.7%), and stroke in 14 patients (4.0%). There were no significant differences in hospital mortality and postoperative complications between the two groups except for renal failure requiring hemodialysis (Non-TR group 6.3%, TR group 22.6%, p < 0.01).

In terms of hemodynamic parameters at two weeks after transplantation, the TR group had significantly higher CVP (8.0 ± 4.4 vs. 10.8 ± 5.5, p < 0.01), mean PAP (24.3 ± 7.2 vs. 27.6 ± 6.6, p = 0.02), PAWP (15.4 ± 5.6 vs. 18.5 ± 5.6, p < 0.01) compared to the non-TR group.

### Pacemaker implantation

Four patients (1.1%) required pacemaker implantation within one month after transplantation: two patients (1.4%) for sick sinus node dysfunction, one patient (0.3%) for complete atrioventricular block, and one patient (0.3%) for left bundle branch block.

### Late clinical outcomes

Late clinical outcomes are shown in Table 3. In-hospital mortality cases were excluded. No patients developed tricuspid valve stenosis that required intervention. During the follow-up period, patients in TR group required hemodialysis more frequently and had high-grade rejection (grade 2 or higher) compared to non-TR group. Based on the latest hemodynamic assessment (mean 22.0 ± 11.3 months), the TR group had significantly higher CVP (6.9 ± 4.6 vs. 10.3 ± 8.7, p < 0.01), mean PAP (22.1 ± 6.7 vs. 28.1 ± 12.0, p < 0.01), PAWP (12.9 ± 5.0 vs. 17.9 ± 10.4, p < 0.01), and lower cardiac index (CI) (3.21 ± 0.7 vs. 2.77 ± 0.7, p < 0.01).

**Table 3:** Late clinical outcomes

Variables	All (n = 338)	Non-TR group (n = 307)	TR group (n = 31)	p
Hemodialysis	34 (10.1%)	24 (7.8%)	10 (32.3%)	< 0.01
The number of times of biopsies	15.1 ± 6.5	14.9 ± 6.2	17.7 ± 8.1	0.02
Rejection ≧ grade 2	105 (31.1%)	88 (28.7%)	17 (54.8%)	< 0.01
CVP (mmHg)	6.9 ± 4.6	6.6 ± 3.8	10.3 ± 8.7	< 0.01
Mean PAP (mmHg)	22.7 ± 7.5	22.1 ± 6.7	28.1 ± 12.0	< 0.01
PAWP (mmHg)	13.4 ± 5.9	12.9 ± 5.0	17.9 ± 10.4	< 0.01
PAPi	3.61 ± 3.2	3.62 ± 3.2	3.54 ± 2.9	0.90
CVP/PAWP	0.53 ± 0.7	0.54 ± 0.7	0.52 ± 0.2	0.97
CI (L/min/m^2^)	3.17 ± 0.7	3.21 ± 0.7	2.77 ± 0.7	< 0.01

CVP: central venous pressure, PAP: pulmonary artery pressure, PAWP: pulmonary artery wedge pressure, CI: cardiac index.

### Occurrence of significant TR

**Fig. 1:**
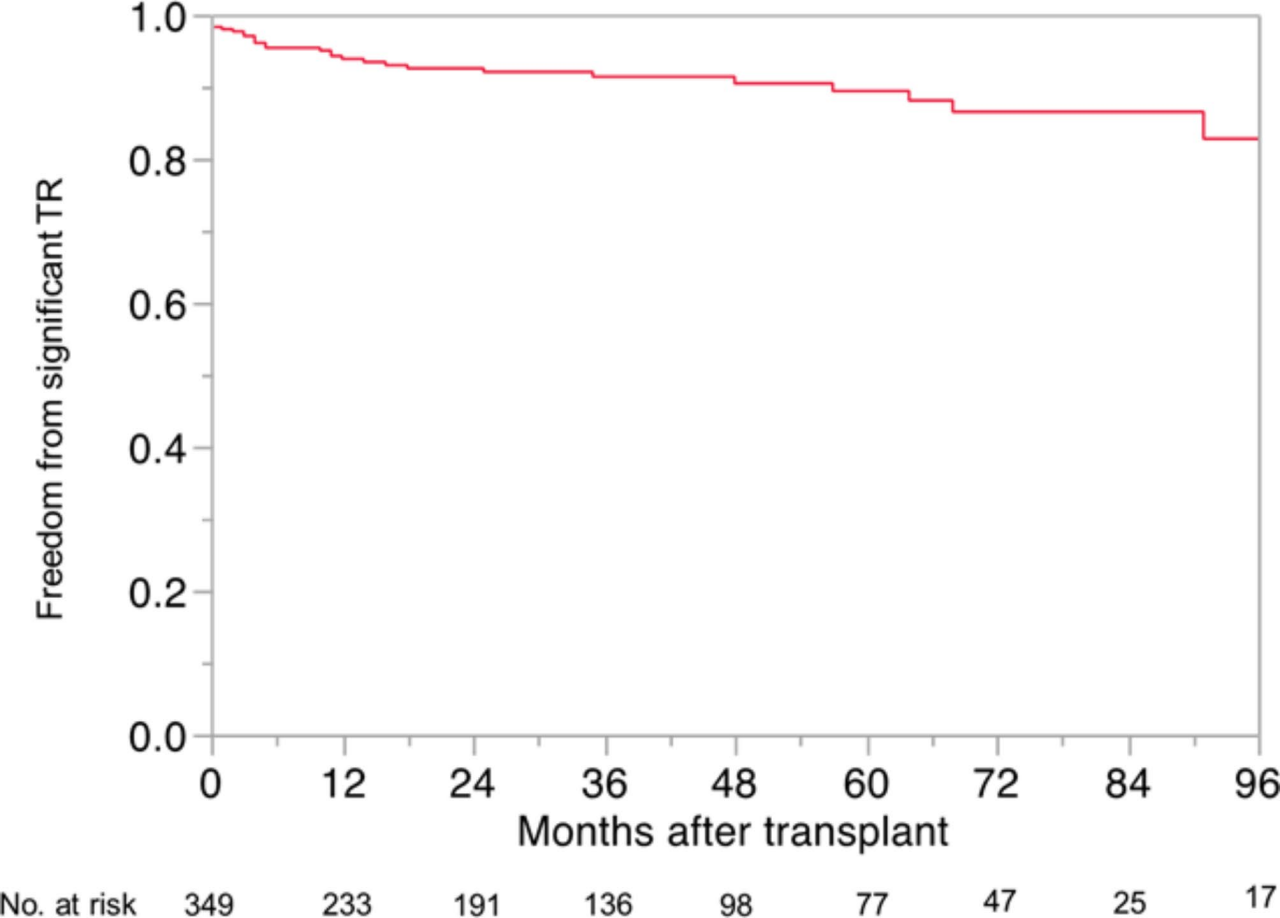
Freedom from significant TR

Thirty-one patients (8.9%) developed significant TR during follow-up. The rate of freedom from significant TR was 93.8% at 1 year, 92.5% at 3 years, and 89.4% at 5 years (Fig. 1). Of note, there were only ten patients (2.9%) who developed persistent significant TR.

### Long-term survival

**Fig. 2:**
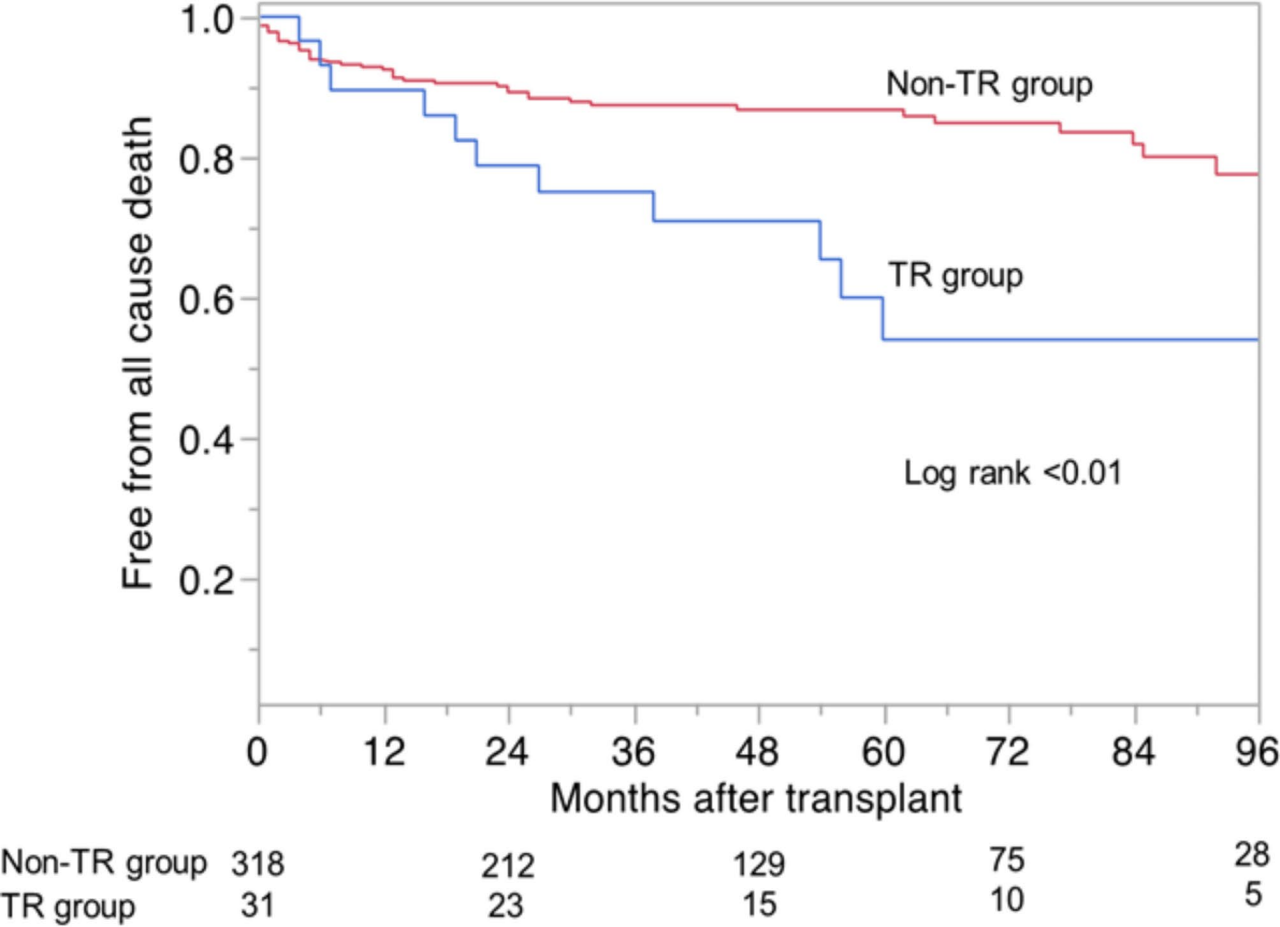
Freedom from all cause death of the TR and non-TR groups. The Kaplan-Meier survival estimates and log-rank test have shown a lower long-term survival rate in the TR group than in the non-TR group

Overall, the actuarial survival rate was 92.6% at 1 year, 86.2% at 3 years, and 83.8% at 5 years. The survival rate of patients in TR group was significantly lower than that of those in non-TR group (5-year survival 54.6% in TR group, 86.7% in non-TR group; log rank < 0.01) (Fig. 2). In TR group, 11 out of 31 patients expired during follow-up. The causes of death in TR group were heart failure (n = 4), respiratory failure (n = 2), septic shock (n = 1), sudden cardiac arrest (n = 1), pulmonary embolism (n = 1), and unknown (n = 2). The Cox proportional hazard regression analysis revealed that significant TR (hazard ratio 2.92, 95% CI 1.47–5.82, p < 0.01) was associated with long-term mortality (Table 4).

**Table 4:** The impact of significant TR on freedom from all cause death

Risk factors	Hazard ratio	95% confidence interval	p
Significant TR	2.92	1.47–5.82	< 0.01
Age	1.01	0.98–1.03	0.70
Rejection ≧ grade 2	0.80	0.44–1.45	0.46
Diabetes mellitus	1.55	0.89–2.73	0.12

## Discussion

This study demonstrated three main findings. First, the occurrence of significant TR after heart transplantation in this study (8.9%) was significantly less frequent than that in previously reported series (20–40%) [1–4]. Regarding persistent significant TR, the incidence was even lower (2.9%). The prophylactic donor heart tricuspid valve annuloplasty might be a durable technique to reduce the incidence of long-term significant TR. Second, the prophylactic donor heart tricuspid valve annuloplasty was safely performed with very low incidence of pacemaker implantation for complete atrioventricular block and no tricuspid valve stenosis. Third, significant TR developed in the patients with prophylactic annuloplasty had a negative impact on the long-term survival. Multivariate analysis revealed that significant TR was an independent predictor of long-term mortality.

### Tricuspid regurgitation after heart transplantation

Although the definition of significant TR is different among published articles, it has been documented that significant TR could occur in the range of 20–40% after OHT and it negatively affects clinical outcomes [1–4]. Aziz and colleagues reported that the occurrence of moderate or greater TR at 12 months after heart transplantation was 35.7% [1]. Bishawi and colleagues demonstrated that 21% of their patients who underwent heart transplantation experienced moderate or greater TR immediately after transplant [2]. Chan and colleagues showed that 33.6% patients experienced moderate or greater TR during their follow up period (43 ± 38 months) [4]. Chen and colleagues also reported that 26.4% developed moderate to severe TR during 66 months [5]. In an effort to reduce the incidence of TR, a number of strategies have been implemented [1, 2, 8–10]. The bicaval anastomosis technique is one of them that secondarily brought a benefit to reduce the incidence of significant TR. Zijderhand and colleagues reported that late tricuspid regurgitation was less frequently seen in patients with bicaval anastomosis (incidence of significant TR 28.5%, rate ratio 2.14, 95% CI 1.17–3.94, p = 0.014) [13]. Prophylactic tricuspid annuloplasty is also another important strategy that would directly contribute to preventing TR. Only a limited number of studies are available in the literature [8, 9]. Greenberg and colleagues recently reported that the incidence of significant TR after 6 months of OHT with prophylactic tricuspid annuloplasty was 1.3% and that without prophylactic annuloplasty was 9.3% [8]. We performed prophylactic tricuspid annuloplasty using the De Vega procedure in donor hearts routinely and demonstrated that the incidence of significant TR during follow-up period was 8.9% during significantly longer follow-up period (43.8 ± 32.9 months). In addition, the incidence of persistent significant TR was almost negligible. Comparing previously published studies, the incidence of significant TR in our study is lower and it seems that the prophylactic annuloplasty strategy has had a preventive effect [1–4].

### Significant tricuspid regurgitation negatively affected long-term survival after heart transplantation

This study demonstrated that patients with significant TR had worse hemodynamic parameters and more renal failure that required hemodialysis compared to those without significant TR. In addition, significant TR was an independent predictor of long-term mortality for all cause death. However, this study did not show cause and effect but rather a relationship between significant TR and poor outcomes.

A number of studies have described that TR after OHT was related with serious clinical conditions including right heart failure, peripheral edema, ascites, renal dysfunction, and death [1, 2, 8–10]. Bishawi and colleagues reported that TR after heart transplantation was associated with renal dysfunction and long-term survival (log rank p < 0.001) [2]. Anderson and colleagues also reported that even mild or greater TR at the time of transplantation predicted poor late survival (long rank p < 0.001) [10]. Moreover, several investigators also demonstrated the similar results that TR had negative impact on long term survival in cardiac surgery [14, 15]. Given the fact that are supported by those reports, it is important to prevent the occurrence of TR after OHT, which in turn contribute to improving clinical outcomes. In the light of the effectiveness of prophylactic TVA with the minimal disadvantages, we believe that prophylactic DeVega annuloplasty is recommended in all cases at the time of OHT.

### What is the optimal surgical technique for prophylactic tricuspid annuloplasty in OHT?

It would be a consensus that ring annuloplasty is preferable rather than suture annuloplasty in the setting of treating TR in “regular” cardiac cases. Sohn and colleagues reported long-term outcomes of ring annuloplasty versus suture annuloplasty [16]. The rate of recurrent TR was higher in suture annuloplasty group (11.9%) than in ring annuloplasty group (1.5%) while there was no difference in cardiac death, pacemaker implantation, and tricuspid valve reoperation. However, different considerations would be necessary when it comes to a prophylactic tricuspid annuloplasty in donor hearts during OHT which generally have no TR. It is debatable to apply the ring annuloplasty technique in hearts that have normal anatomy. The ring annuloplasty technique uses an artificial material (i.e. ring), which could increase the risk of infection in immunosuppressed recipients. In addition, there are concerns of cost as well as a certain degree of increasing ischemic time. Therefore, we chose the DeVega suture annuloplasty technique to prevent TR exclusively in all OHT cases. It is simple, quick, cost effective, and using minimal foreign materials preserves physiologic valve architecture [9, 17–23]. Not only the prophylaxis purpose of TR, but also it was expected to decrease the incidence of acute right ventricle failure in the acute phase after heart transplantation. Malinowski and colleagues reported that the DeVega annuloplasty successfully treated tricuspid regurgitation and preserved normal annular dynamics and geometry during acute right heart failure condition in an ovine preparation [21]. We believe the DeVega suture annuloplasty would be the best option in OHT.

### Pacemaker implantation after heart transplantation

Some patients may require a pacemaker implantation after heart transplantation. Cantillon and colleagues reported that pacemaker implantation occurred in 10.9% during the follow-up period of 6.3 years after OHT based on the database of United Network for Organ Sharing/Organ Procurement and Transplantation Network (UNOS/OPTN) [24]. In the present study, the rate of pacemaker implantation was overall 2.3% during 43.8 ± 32.9 months. This is comparable or slightly better compared to them. Especially, at the time of DeVega annuloplasty, surgeons should to be mindful of the risk of atrioventricular conduction system injury. Rubin and colleagues reported that pacemaker implantation for complete atrioventricular block was required in 4.0% after the prophylactic DeVega annuloplasty in heart transplantation [25]. While the incidence that required pacemaker implantation in the present study was low, it is important to note that, as long as the DeVega annuloplasty procedure is added, there is always a potential risk of injuring the conduction system. Of note, we experienced only one patient (0.3%) who required a pacemaker implantation for complete atrioventricular block. We believe that the prophylactic DeVega annuloplasty can be performed safely at the time of heart transplantation.

### Limitations

There are some limitations that need to be addressed. First, this is a retrospective and single-center study. A prospective randomized study would be warranted to validate the findings of this study. Second, the sample size and follow-up period in the cohort was somewhat limited. Therefore, the statistical power might be limited. Third, since TVA was exclusively performed in all cases, this study lacks a control group (i.e. OHT without TVA). Therefore, this study did not describe if there were any increased risks of complications (e.g. complete heart block) by adding a TVA.

## Conclusion

Prophylactic donor heart tricuspid valve annuloplasty has the potential to reduce the occurrence of significant TR and can be performed safely. Significant TR developed in the patients with prophylactic annuloplasty negatively affected the long-term survival and was an independent predictor of long-term mortality.

## Data Availability

Data available on request.

## Abbreviations

TR: Tricuspid regurgitation
OHT: Orthotopic heart transplantation
TVA: Tricuspid valve annuloplasty
PAPi: Pulmonary artery pressure index
CVP: Central venous pressure
PAWP: Pulmonary artery wedge pressure
EF: Ejection fraction
ECMO: Extracorporeal membranous oxygenation
CI: Cardiac index
LVAD: Left ventricular assist device
LVDd: Left ventricular end-diastolic diameter
